# Urine concentration ability is reduced to the same degree in adult dominant polycystic kidney disease compared with other chronic kidney diseases in the same CKD-stage and lower THAN in healthy control subjects - a CASE control study

**DOI:** 10.1186/s12882-020-02043-w

**Published:** 2020-08-31

**Authors:** M. H. Malmberg, F. H. Mose, E. B. Pedersen, J. N. Bech

**Affiliations:** University Clinic in Nephrology and Hypertension, Regional Hospital West Jutland and University of Aarhus, Holsstebro, Denmark

**Keywords:** Aldosterone, Angiotensin II, aquaporin2, Blood pressure, Chronic kidney disease, Epithelial sodium channel, GFR, Osmolality, Polycystic kidney disease, Renin, Sodium, Vasopressin

## Abstract

**Background:**

Concentration of the urine is primarily regulated via vasopressin dependent aquaporin-2 water channels in the apical membrane of kidney principal cells. It is unclear whether urine concentration ability in ADPKD differs from other patients with similar degree of impaired renal function (non-ADPKD patients). The purpose of this case control study was to measure urine concentration ability in ADPKD patients compared to non-ADPKD patients and healthy controls.

**Methods:**

A seventeen hour long water deprivation test was carried out in 17 ADPKD patients (CKD I-IV), 16 non-ADPKD patients (CKD I-IV), and 18 healthy controls. Urine was collected in 4 consecutive periods during water deprivation (12 h, 1 h, 2 h and 2 h, respectively) and analyzed for osmolality (u-Osm), output (UO), fractional excretion of sodium (FE_Na_), aquaporin2 (u-AQP2) and ENaC (u-ENaC). Blood samples were drawn trice (after 13-, 15-, and 17 h after water deprivation) for analyses of osmolality (p-Osm), vasopressin (p-AVP), and aldosterone (p-Aldo).

**Results:**

U-Osm was significantly lower and FE_Na_ significantly higher in both ADPKD patients and non-ADPKD patients compared to healthy controls during the last three periods of water deprivation. During the same periods, UO was higher and secretion rates of u-AQP2 and u-ENaC were lower and at the same level in the two groups of patients compared to controls. P-AVP and p-Osm did not differ significantly between the three groups. P-Aldo was higher in both groups of patients than in controls.

**Conclusions:**

Urine concentration ability was reduced to the same extent in patients with ADPKD and other chronic kidney diseases with the same level of renal function compared to healthy controls. The lower urine excretion of AQP2 and ENaC suggests that the underlying mechanism may be a reduced tubular response to vasopressin and aldosterone.

**Trial registration:**

Current Controlled Trial NCT04363554, date of registration: 20.08.2017.

## Background

Urine volume is primarily regulated by vasopressin and aquaporin-2 water channels in the apical membrane of principal cells in the kidney [1]. The renin-angiotensin-aldosterone system, the natriuretic peptide system and the sympathetic adrenergic nervous system also play a role in the opening degree of the AQP2 channels and therefore affect urine concentration [2–7]. The ability to concentrate urine can be measured by a water deprivation test. Generally, urine concentration is reduced in kidney diseases, but the underlying mechanisms and the severity may deviate in different kidney disease, although the degree of renal impairment is the same [8, 9].

Autosomal dominant polycystic kidney disease (ADPKD) is a common genetic disease, which is characterized by the formation of cysts in the kidneys, leading to renal impairment [10]. Previous studies indicated that vasopressin is a significant factor in the pathogenesis and progression of the disease by stimulating the growth of kidney cysts [11–14]. Meijer et al. showed that in an early stage of ADPKD, patients had an increased urine output and decreased urine osmolality compared to healthy controls [15]. Zittema et al. suggested that ADPKD patients had a decreased urine concentration ability, higher plasma osmolality, and higher plasma vasopressin compared to healthy individuals [16]. In a previous study, ADPKD patients had an impaired urine concentration ability compared to patients with IgA-nephropathy, and it was suggested that urine concentration ability was impaired to a larger extent during progressive renal disease in ADPKD than in other chronic kidney diseases [17]. However, it is unclear whether urine concentration ability in ADPKD differs from patients with impaired renal function due to other causes (non-ADPKD patients).

The purpose of the present case-control study was to perform a urine concentration test in patients with ADPKD (Group1), in patients with chronic kidney diseases without ADPKD (Group 2) and in healthy control subjects (Group 3). We measured:1) Urine osmolality (u-Osm), urine output (UO), free water clearance (C_H20_), fractional excretion of sodium (FE_Na_).

2) Urine excretion of aquaporin 2 water channels (u-AQP2) and urine excretion of a fraction of the epithelial sodium channels (u-ENaC), 3) Plasma concentrations of vasopressin (p-AVP), renin (PRC), angiotensin II (p-AngII) and aldosterone (p-Aldo), and plasma osmolality (p-Osm), 4) Blood pressure (BP), augmentation index (AIx) and Pulse wave velocity (PWV).

We hypothesized that ADPKD patients have decreased urinary concentration ability compared to non-ADPKD patients with chronic kidney disease, as well as compared to healthy controls. We also hypothesized that a difference in urinary concentration ability between ADPKD and non-ADPKD and the control group may be explained by a change in the renal tubular response and/or changes in vasoactive hormones.

## Methods

### Study design

The design was as a case-control study with three groups. Group 1 comprised patients with ADPKD, Group 2 comprised patients with chronic kidney diseases other than ADPKD, and Group 3 comprised healthy control subjects. Each subject was examined once.

### Recruitment

Participants was recruited in the period between September 2017 and November 2018. In Groups 1 and 2, patients were recruited from the Outpatient Clinic of Nephrology in University Clinic in Nephrology and Hypertension, Department of Medicine at Holstebro Hospital, Denmark. Healthy controls in Group 3 were recruited by advertising in the local newspaper. All three groups were matched regarding age and gender. The two patient groups were matched regarding GFR.

### Subjects

#### Inclusion criteria

##### Group 1

Patients with ADPKD. Age 18 years or older. Men and unfertile women or fertile women using safe contraception throughout the trial period (safe contraception was defined as: sterilization, birth control pills, spiral, subdermal implantation, hormonal vaginal ring, transdermal patch, depot injection of progestogen or sexual abstinence). Kidney function corresponding to chronic kidney disease (CKD) stages I-IV (eGFR > 15 mL/min/1.73 m2). Patients were included if a genetic test revealed PKD1 and PKD2 mutations. If a genetic test was not performed, patients were included in accordance with the classic Ravine criteria using ultra- sonographic findings. Thus, ADPKD patients were diagnosed by genetic testing for PKD1 and PKD2 mutations, or presence of one of the following ultra- sonographic findings in accordance to the classic Ravine criteria. Patients with a negative family history of ADPKD with more than 10 cysts in each kidney, and with exclusion of other causes of extra renal or renal cyst formations using medical history, clinical examination and CT- and/ or MR-scanning. In addition, patients with a family history of ADPKD: 18–39 yrs. with 3 cysts or more uni- or bilaterally/ 40–59 yrs. with 2 or more cysts bilaterally/ ≥60 yrs. with at least 4 cysts bilaterally [18]. *Group 2*: Same criteria as for Group 1, but without criteria for ADPKD. *Group 3*: Age 18 years or older. Healthy men and unfertile women or fertile women using safe contraception throughout the trial period (as indicated for Group 1).

#### Exclusion criteria

##### Groups 1 and 2

Previous kidney transplantation. Previous kidney operation. Diabetes mellitus. Lithium nephropathy. Medullary cystic kidney disease. Neoplastic disease. Pregnancy or breastfeeding. Withdrawn consent. Intolerance to or unacceptable side effects to water deprivation test. Alcohol or drug abuse. Blood pressure > 170/110 mmHg despite treatment with metoprolol and/or amlodipine. *Group 3*: Arterial hypertension i.e. office blood pressure above 140 mmHg systolic and / or 90 mmHg diastolic. Significant clinical signs of heart disease, diseases of the lungs, liver, kidneys, endocrine organs, the brain or neoplastic disorders. Alcohol or drug abuse. Medical treatment except oral contraceptives. Smoking. Pregnancy or breastfeeding. Clinically significant abnormal findings in blood tests (plasma concentration of sodium, potassium, albumin, creatinine, bilirubin, alanine-amino-transaminase (ALAT), alkaline phosphatase, cholesterol, calcium, phosphate, parathyroid hormone (PTH), thyroid-stimulating hormone (TSH), blood concentration of hemoglobin, leukocytes, platelets and glycosylated hemoglobin A1c (HbA1c), or in the urine sample (leukocytes, nitrite, blood, glucose, albumin). Clinically significant changes in electrocardiogram. Blood donation within the last month before the test date in the first trial sequence. Intolerance to or unacceptable side effects to water deprivation test.

#### Withdrawal criteria

Development of exclusion criteria. Serious or unacceptable side effects.

### Effect variables

The primary effect variable was u-osmolality (u-Osm) during thirst_._ The secondary effect variables were p-AVP, u-AQP-2, p-Osm, UO, C_H20_, fractional excretion of sodium (FE_Na_), u-ENaC, creatinine clearance (C_Cre_**),** brachial blood pressure (bBP), central blood pressure (cBP), PWV, AIx, PRC, p-AngII and p-Aldo.

### Number of subjects

With a minimal relevant difference of 145 mOsm/kg in u-Osm with an estimated standard deviation (SD) of 187 mOsm/kg, 16 subjects were needed using a level of significance of 5% and a statistical power of 90%. To counteract any dropouts, we included 20 participants from each group.

### Antihypertensive medications

Antihypertensive medications including diuretics, angiotensin-converting enzyme inhibitors and angiotensin-II inhibitors were discontinued or substituted with metoprolol 25 mg and/or amlodipine 5 mg 14 days prior to each test day. Throughout the study period, bBP was monitored using an oscillometric home blood pressure monitor. At a blood pressure of > 170/110 mmHg, metoprolol 25 mg and/or amlodipine 5 mg was given and increased up to metoprolol 100 mg and / or amlodipine 10 mg. Subjects were discontinued from the study if blood pressure above 170/110 mmHg continued despite treatment with metoprolol 100 mg and/ or amlodipine 10 mg. Immediately after the examination day ended, the usual antihypertensive treatment was resumed.

### Ethics

Regional Committee on Health Research Ethics approved the study (case number: 1–10–72-147-17). The study was done in agreement with the Declaration of Helsinki and was registered at clinicaltrials.gov***(identifier:*** ***NCT04363554******).*** Written informed consent was obtained from each subject.

### Experimental procedure

The study took place at the Laboratory in the University Clinic in Nephrology and Hypertension. On the day prior to the examination day, all subjects consumed their habitual intake of food and beverage, but no alcohol until 07.30 PM. Thereafter, the subjects fasted and thirsted during 12 h before arriving at the Laboratory in the University Clinic in Nephrology and Hypertension. Measurements took place in a quiet and temperature-controlled room (22–25 °C), and the subjects were in a supine position. The central and brachial blood pressure was measured every 20 min by Mobil-O-Graph® PWA mounted on the upper arm. In the other arm an intravenous catheter was placed to collect blood samples. Urine samples were collected by voiding in standing or sitting position after blood samples had been collected. The subjects were weighed at arrival and every two hours during the examination. During the water deprivation test, urine was collected during 12 h from 0730 PM the day before arrival to the laboratory to 0730 AM on the examination day (Period 1). In addition, urine was collected in three periods: 0730 AM to 0830 AM (Period 2), 0830 AM to 1030 AM (Period 3), and 1030 AM to 1230 PM (Period 4). Urine samples were taken for measurements of u-Osm, u-AQP-2 and u-ENaC, u-Na, u-K, u-albumin (u-Alb), u-creatinine. Blood samples were drawn at the end of Periods 2, 3 and 4 for determination of p-AVP, p-Osm, PRC, p-AngII, p-Aldo, plasma creatinine, p-Na, p-K, and p-alb.

### Measurements

#### Renal function

The CKD-EPI formula was used to calculate estimated GFR (eGFR). Clearance (C) of substance X was calculated as C_X_ = U_X_ * UO/P_X_), where UO is urine excretion rate, U_X_ denotes concentration of x in urine, while P_X_ denotes concentration of x in plasma. FE_Na_ was calculated with the formula: FE_Na_ = 100 * ((u-Na * p-Cr)) / ((p-Na * u-Cr)). Free water clearance was calculated with the formula: C_H2O_ = UO-C_osm._

#### AQP2 and ENaC in urine

At − 20 °C, urine samples were kept frozen until assayed. As previously described, u-AQP2 was determined by radioimmunoassay (RIA) [19, 20]. Professor Soren Nielsen and Professor Robert Fenton (The Water and Salt Research Center, Aarhus University, Denmark) provided with rabbit anti-AQP2 antibodies. The minimal detection level was 32 pg/tube, inter-assay was 11.7% and the intra-assay 5.9%. As described previously u-ENaCγ was measured by RIA [21, 22]. Lofstrand, Gaithersburg, Maryland, USA synthesized the ENaCγ. Professor Soren Nielsen and Professor Robert Fenton (The Water and Salt Research Center, Aarhus University, Denmark) provided with the ENaCγ antibody. Minimal detection level was 35 pg/tube, inter-assay was 10% at a mean level of 338 pg/tube, 9% at a mean level of 743 pg/tube, and intra-assay was 5.0% in the range 125–135 pg/tube and 5.6% in the range 290–380 pg/tube.

#### Vasoactive hormones in plasma

We centrifuged the blood samples for measurements of vasoactive hormones, for 10 min at 2200 G and 4^°^C. The plasma was thereafter separated from blood cells and kept frozen until assayed. As previously described, p-AngII and p-AVP were extracted from plasma with C_18_ Sep-Pak (Waters Corporation, Milford, MA, USA) and determined by RIA [23, 24]. Professor Jacques Dürr (Miami, FL, USA) provided with the antibodies against AVP. Minimal detection level was 0.5 pmol/L, inter-assay was 13% and intra-assay was 9%. From the Department of Clinical Physiology, Glostrup Hospital, Denmark antibodies against AngII were obtained. Minimal detection level was 2 pmol/L, inter-assay was 12% and intra-assay was 8%. A RIA kit from Demeditec Diagnostics GmbH (Kiel, Germany) was used to determined p-Aldo. Minimal detection level was 14.8 pg/mL, inter-assay was 10.2% and intra-assay was 11.9%. A RIA kit from CIS Bio International (Gif-Sur-Yvette Cedex, France) was used to determined PRC. Minimal detection level was 1 pg/mL, inter-assay was 4.1% and intra-assay was 1.8%.

#### Other biochemical measurements

A_2_O Advanced Automated Osmometer (Advanced Instruments, MA, USA) was used to measure u-Osm and p-Osm. Plasma concentration of sodium, potassium, albumin, hemoglobin, leukocytes, platelets, creatinine, bilirubin, ALAT, alkaline phosphatase, cholesterol, calcium, phosphate, PTH, TSH and HbA1c were measured using routine methods at the Department of Clinical Biochemistry (Holstebro Hospital, Denmark).

#### Brachial and central blood pressure

BP was measured every twenty minutes throughout the examination day. Heart rate, bBP, cBP, mean arterial pressure (MAP), PWV and AIx was measured using an oscillometric device (Mobil-O-Graph® PWA).

#### Statistics

IBM SPSS statistics version 20 (SPSS Inc., Chicago, IL, USA) was used to perform statistical analyses. For comparison between and within the three groups, we used A General Linear Model with Greenhouse-Geisser correction for violating the assumption of sphericity with repeated-measures ANOVA. Data were tested for normal distribution. For comparison between two groups, we used a paired or unpaired t-test, when data showed normal distribution. For data which did not show normal distribution, we used Mann-Whitney’ s U test for unpaired data, and Wilcoxon’s Signed Rank Test for paired data. Statistical significance was set at < 0.05 in all analyses. Data with normal distribution are reported as means ± SD and data with non-normal distribution are reported as medians with 25 and 75% percentiles in brackets.

## Results

### Demographics

In Table 1, baseline demographics and clinical characteristics are presented. Twenty ADPKD patients with chronic kidney disease stages I-IV were allocated to the study. All ADPKD patients were included according to the classic Ravine criteria [18]. One patient was excluded due to cannulation problems. Two patients were excluded due to withdrawal of consent. Thus, 17 patients were included in Group 1. The distribution of these patients in different CKD stages is as follows: CKD stage 1 (*n* = 4), CKD stage 2 (*N* = 8), CKD stage 3a (*n* = 3), CKD stage 3b (*n* = 1), CKD stage 4 (n = 1). Twenty non-ADPKD patients with CKD stage I-IV were allocated to the study. One patient was excluded due to side effects of the background antihypertensive medicine. Three patients were excluded due to withdrawal of consent. Thus, 16 patients were included in Group 2. The distribution of these patients in different CKD stages is as follows: CKD stage 1 (*n* = 4), CKD stage 2 (*N* = 4), CKD stage 3a (*n* = 2), CKD stage 3b (n = 2), CKD stage 4 (n = 4). Twenty healthy controls were included in the study. Two subjects were excluded due to withdrawal of consent. Thus, 18 subjects were included in Group 3.

**Table 1:** Baseline demographics of the participants in the study. Patients with adult dominant polycystic kidney disease (ADPKD), patients with non-ADPKD kidney disease (Non-ADPKD), and healthy control subjects (Controls)

	ADPKD	Non-ADPKD	Controls
Number of subjects (n)	17	16	18
Age (years)	53 [44;63]	56 [43;71]	57 [54;66]
Gender (men/women)	8 /9	9 /7	8 /10
Office systolic brachial BP (mmHg)	138 ± 16*	130 ± 16	125 ± 10
Office diastolic brachial BP (mmHg)	79 ± 11#	72 ± 9	74 ± 7
eGFR (ml/min/1.73m^2^)	71 ± 26*	63 ± 33*	87 ± 13
P-Creatinine (μmol/l)	101 ± 38*	124 ± 57*	76 ± 11
U-Albumin (mg/l)	14 [8;53]*#	81 [26;283]*	8 [3;11]

Values represent n in either group or mean ± SD or median with 25 and 75% percentiles in brackets. * = *p* < 0.05 between ADPKD or non-ADPKD patients and healthy controls. # = p < 0.05 between ADPKD and non-ADPKD patients

Al three groups had similar age (ADPKD patients had a median age of 53 years, non-ADPKD patients 56 years, healthy controls 57 years, *p* = 0.557 between ADPKD and non-ADPKD patients, *p* = 0.503 between ADPKD patients and healthy controls and *p* = 0.878 between non-ADPKD patients and healthy controls). ADPKD patients and non-ADPKD patients had similar eGFR (71 ± 26 ml/min/17.3m^2^ and 63 ± 33 ml/min/1.73m^2^, *p* = 0.433), but both patient groups had a lower eGFR compare to healthy controls (87 ± 13 ml/min/1.73m^2^, *p* = 0.026 and *p* = 0.011, respectively). ADPKD patients´ antihypertensive treatment before this present study were: angiotensin II-receptor blockers (*n* = 7), ACE-inhibitors (n = 7), calcium-antagonists (*n* = 6), loop diuretics (*n* = 2), thiazides (*n* = 4), beta-adrenergic blockers (n = 4), alfa-blockers (*n* = 1) and potassium-sparing diuretics (n = 1). Non-ADPKD patients´ antihypertensive treatment before this present study were: angiotensin II-receptor blockers (*n* = 5), ACE-inhibitors (n = 4), calcium-antagonists (n = 4), loop diuretics (*n* = 2) and beta-adrenergic blockers (*n* = 3). Among ADPKD patients, 11 received metoprolol, 13 amlodipine, and 2 no antihypertensive treatment during the study period. Among non-ADPKD patients, 5 received metoprolol, 5 amlodipine, and 6 no antihypertensive treatment during the study period. Overall, more antihypertensive treatment was needed in the ADPKD group. In Group 1, 2 patients had a negative family story. In Group 2, 10 patients had a renal biopsy and 6 patients had no biopsy. The primary kidney disease was chronic non-specified glomerulonephritis in 5 patients, chronic interstitial nephritis in 2 patients, focal segmental glomerulosclerosis (FSGS) in 3 patients. The patients in Group 2 had a stable kidney function for more than 3 months and none received immunosuppressive therapy.

### Renal water excretion

Table 2 shows u-Osm, UO, C_H2O_, and u-AQP2, and Table 4 shows p-Osm and p-AVP.

**Table 2:** U-osmolality (U-Osm), urine excretion of aquaporin2 (u-AQP2), urine output (UO), and free water clearance (C_H2O_) during a water deprivation test in patients with adult dominant polycystic kidney disease (ADPKD), patients with non-ADPKD kidney disease (Non-ADPKD), and healthy control subjects (Controls). Urine was collected in four periods. Period 1 (twelve hours): from 0730 PM the day before arrival to the laboratory to 0730 AM on the examination day. Period 2 (one hour): 0730 to 0830 AM. Period 3 (two hours): 0830–1030 AM. Period 4 (two hours): 1030–1230 AM

	Period 1 07.30 PM- 07.30 AM	Period 2 07.30 AM- 08.30 AM	Period 3 08.30 AM–10.30 AM	Period 4 10.30 AM- 12.30 PM
U-Osm (mosmol/kg)				
ADPKD	498 ± 149	518 ± 143*	491 ± 133*	569 ± 105*
Non-ADPKD	496 ± 220	603 ± 143*	532 ± 192*	579 ± 114*
Controls	620 ± 233	765 ± 150	694 ± 198	757 ± 167
Comparison within groups	0.339			
Comparison between groups	< 0.001			
UO (ml/min)				
ADPKD	1.6 ± 0.7	1.3 ± 0.5*	1.8 ± 1.1*	1.3 ± .5*
Non-ADPKD	1.5 ± 0.6	1.0 ± 0.3*	1.6 ± 0.7*	1.2 ± 0.4*
Controls	1.1 ± 0.6	0.7 ± 0.3	1.0 ± 0.5	0.9 ± 0.3
Comparison within groups	0.736			
Comparison between groups	0.001			
C_H2O_ (ml/min)				
ADPKD	−0.8 ± 0.5	−0.9 ± 0.5	−0.9 ± 0.8	−1.1 ± 0.4
Non-ADPKD	− 0.8 ± 0.7	−1.0 ± 0.5	−0.9 ± 0.5	−1.1 ± 0.4
Controls	− 0.9 ± 0.5	−1.1 ± 0.5	−1.1 ± 0.4	−1.3 ± 0.3
Comparison within groups	0.560			
Comparison between groups	0.482			
U-AQP2 (ng/ml)				
ADPKD	0.8 ± 0.3	0.9 ± 0.4*	0.7 ± 0.3*	0.8 ± 0.2*
Non-ADPKD	0.8 ± 0.6	1.0 ± 0.5*	0.7 ± 0.4	0.8 ± 0.2*
Controls	1.1 ± 0.5	1.5 ± 0.6	1.1 ± 0.7	1.1 ± 0.4
Comparison within groups	0.407			
Comparison between groups	0.001			
AQP2 (ng/min)				
ADPKD	0.6 [0.2;0.8]	0.7 [0.4;1.5]*	0.5 [0.3;0.9]*	0.6 [0.3;0.9]*
Non-ADPKD	0.4 [0.2;0.6]*	1.0 [0.6;1.6]*	0.5 [0.2;1.0]*	0.7 [0.5;1.0]*
Controls	0.7 [0.2;0.6]	2.1 [1.5;3.8]	1.5 [1.1;2.1]	1.4 [1.0; 2.2]
Kruskal-Wallis test	0.097	0.001	0.003	0.002

A General Linear Model with Greenhouse-Geisser correction was used for comparison within and between the three groups. Values represent mean ± SD or median with 25 and 75% percentiles in brackets * = p < 0.05 between ADPKD or non-ADPKD- patients and healthy controls. # = *p* < 0.05 between ADPKD and non-ADPKD- patients

Comparisons between the three groups showed no significant differences in u-Osm during Period 1 (ADPKD patients: 498 mosmol/kg, non-ADPKD patients: 496 mosmol/kg, healthy controls: 620 mosmol/kg, *p* = 0.979 between ADPKD and non-ADPKD patients, *p* = 0.074 between ADPKD patients and healthy controls and *p* = 0.122 between non-ADPKD patients and healthy controls). During Period 2, u-Osm was lower in both ADPKD patients (518 mosmol/kg) and non-ADPKD patients (603 mosmol/kg) compared to healthy controls (765 mosmol/kg) with *p* < 0.001 and *p* = 0.006, respectively). These significant differences remained throughout Period 3 and 4 (*p* < 0.05).

There were no differences in UO between the three groups during Period 1 (1.6 ml/min in ADPKD patients, 1.5 ml/min in non-ADPKD patients, and 1.1 ml/min in healthy controls, *p* = 0.832 between ADPKD and non-ADPKD patients, *p* = 0.052 between ADPKD patients and healthy controls, and *p* = 0.057 between non-ADPKD patients and healthy controls). During Period 2, ADPKD patients had an increased UO compare to healthy controls (1.3 ml/min vs. 0.7 ml/min *p* < 0.001), and this difference persisted during Periods 3 and 4 (*p* < 0.05). During period 2, non-ADPKD patients had an increased UO compared to healthy controls during Period 2 (1.0 ml/min vs. 0.7 ml/min, *p* = 0.008), and this difference persisted in Period 3 and 4 (p < 0.05). No differences were seen between ADPKD and non-ADPKD patients during any of the periods (*p* = 0.832 in Period 1, *p* = 0.119 in Period 2, *p* = 0.557 in Period 3 and *p* = 0.468 in Period 4).

C_H2O_ did not differ between the groups (*p* = 0.482)_._

U-AQP2 excretion rate was decreased in non-ADPKD patients compared to healthy controls during Period 1 (0.4 ng/min vs. 0.7 ng/min, *p* = 0.039). During Periods 2, 3 and 4, both ADPKD and non-ADPKD patients had a decreased u-AQP2 excretion rate compared to healthy controls (*p* < 0.05). No significant difference was measured between ADPKD and non-ADPKD patients during Periods 2, 3 and 4 (*p* = 0.339 in Period 2, *p* = 0.708 in Period 3 and p = 0.708 in Period 4).

P-osmolality and AVP did not deviate significantly between the three groups during period 2–4 (*p* = 0.236 and *p* = 0.317, respectively) (Table 4).

Comparison within each group showed no significant changes in u-Osm, UO, C_H2O_, u-AQP2, p-Osm and p-AVP during the test (> 0.05).

### Weight loss and total urine output

Weight loss was similar in the groups during the examination day (2.4 ± 1.0 kg in ADPKD patients, 2.2 ± 1.4 kg in non-ADPKD patients and 2.2 ± 0.9 kg in healthy controls, *p* = 0.634 between ADPKD and non-ADPKD patients, *p* = 0.394 between ADPKD patients and healthy controls and *p* = 0.860 between non-ADPKD patients and healthy controls). However, ADPKD and non-ADPKD patients had a higher urine output during the whole examination compared to healthy controls (1551 ± 485 ml in ADPKD patients, 1475 ± 480 ml in non-ADPKD patients and 1114 ± 445 ml in healthy controls, *p* = 0.654 between ADPKD and non-ADPKD patients, *p* = 0.009 between ADPKD patients and controls, *p* = 0.030 between non-ADPKD patients and controls).

### Renal sodium excretion

Table 3 shows FE_Na_ and u-ENaC.

**Table 3:** Fractional excretion of sodium, (FE_Na_), urine excretion of a fraction of epithelial sodium channel (u-ENaC), urine albumin excretion rate (u-Alb), creatinine clearance (C_Creatinine_) during a water deprivation test in patients with adult dominant polycystic kidney disease (ADPKD), patients with non-ADPKD kidney disease (Non-ADPKD), and healthy control subjects (Controls). Urine was collected in four periods. Period 1 (twelve hours): from 0730 PM the day before arrival to the laboratory to 0730 AM on the examination day. Period 2 (one hour): 0730 to 0830 AM. Period 3 (two hours): 0830–1030 AM. Period 4 (two hours): 1030–1230 AM

	Period 1 07.30 PM- 07.30 AM	Period 2 07.30 AM- 08.30 AM	Period 3 08.30 AM–10.30 AM	Period 4 10.30 AM- 12.30 PM
FE_Na_ (%)				
ADPKD	1.1 ± 0.5*	0.9 ± 0.5*	1.2 ± 0.7*	1.2 ± 0.7*
Non-ADPKD	1.3 ± 0.8*	1.1 ± 0.7*	1.5 ± 1.0*	1.4 ± 1.0*
Controls	0.6 ± 0.3	0.5 ± 0.2	0.7 ± 0.3	0.8 ± 0.3
Comparison within groups	0.910			
Comparison between groups	0.014			
U-ENaC (ng/ml)				
ADPKD	0.6 ± 0.3*	0.6 ± 0.3*	0.4 ± 0.2*	0.4 ± 0.2*
Non-ADPKD	0.6 ± 0.3	0.9 ± 0.5	0.5 ± 0.3*	0.5 ± 0.2*
Controls	0.9 ± 0.4	1.2 ± 0.5	0.8 ± 0.5	0.8 ± 0.3
Comparison within groups	0.394			
Comparison between groups	0.003			
U-ENaC (ng/min)				
ADPKD	0.3 [0.1;0.9]*	0.5 [0.2;1.0]*	0.3 [0.2;0.6]*	0.3 [0.2;0.5]*
Non-ADPKD	0.3 [0.2;0.6]*	0.9[0.5;1.2]*	0.3 [0.1;0.6]*	0.4 [0.3;1.0]*
Controls	0.8 [0.4;2.0]	1.7[1.2;2.9]	1.2 [0.5;2.1]	0.9 [0.7;1.9]
Krusal-Wallis H	0.023	0.001	0.011	< 0.001
U-Alb (mg/min)				
ADPKD	0.01 [0.01;0.02]*	0.02 [0.01;0.05]#	0.01 [0.00;0.03]	0.01 [0.01;0.03]#
Non-ADPKD	0.04 [0.01;0.17]*	0.08 [0.02;0.44]*	0.02 [0.01;0.49]*	0.4 [0.14;0.22]*
Controls	0.01 [0.00;0.01]	0.01 [0.01;0.02)	0.01 [000;0.02]	0.01 [0.00;0.02]
Kruskal-Wallis test	0.003	0.001	0.032	0.001
C_Creatinine_ (ml/min)				
ADPKD	104.0 ± 43.3	109 ± 41	108 ± 36*	106 ± 40*
Non-ADPKD	101.3 ± 57.9	93 ± 50*	101 ± 50*	94 ± 48*
Controls	127.7 ± 27.7	133 ± 28	131 ± 24	132 ± 23
Comparison within groups	0.809			
Comparison between groups	0.040			

A General Linear Model with Greenhouse-Geisser correction was used for comparison within and between the three groups. Values represent mean ± SD or median with 25 and 75% percentiles in brackets * = p < 0.05 between ADPKD or non-ADPKD- patients and healthy controls. # = p < 0.05 between ADPKD and non-ADPKD- patients

Comparisons between groups showed that FE_Na_ was significantly higher in both ADPKD patients (1.1, 0.9, 1.2, and 1.2%, Periods 1–4, respectively) and non-ADPKD patients (1.3, 1.1, 1.5, and 1.4%, Periods 1–4, respectively) compare to healthy controls (0.6, 0.5, 0.7, and 0.8%, Periods 1–4, respectively) throughout the examination day (*p* < 0.05). No difference was seen between ADPKD and non-ADPKD- patients (*p* = 0.394 in Period 1, *p* = 0.328 in Period 2, *p* = 0.372 in Period 3 and *p* = 0.527 in Period 4).

.

U-ENaC excretion rates were similar in ADPKD patients (0.3 ng/min, 0.5 ng/min, 0.3 ng/min, and 0.3 ng/min, Periods 1–4, respectively) and non-ADPKD patients (0.3 ng/min, 0.9 ng/min, 0.3 ng/min, and 0.4 ng/min, Periods 1–4, respectively, *p* > 0.05). The level was significantly lower in both groups of patients compared to healthy controls in all the periods (0.8 ng/min, 1.7 ng/min, 1.2 ng/min, and 0.9 ng/min, Periods 1–4, respectively, *p* < 0.05).

Comparison within each group showed no significant changes in FE_Na_ and u-ENaC during the test.

### Renal albumin excretion and creatinine clearance

Table 3 shows u-Alb and creatinine clearance.

During all Periods non-ADPKD patients had an increased albumin excretion rate compare to healthy controls (*p* = 0.002 in Period 1, *p* < 0.001 in Period 2, *p* = 0.014 in Period 3 and p < 0.001 in Period 4).

Furthermore, non-ADPKD patients had an increased albumin excretion rate in Period 2 and Period 4 compared to ADPKD patients (*p* = 0.008 and *p* = 0.013, respectively). Creatinine clearance was lower in both patient groups compared to healthy controls (*p* < 0.05). Creatinine clearance was the same between ADPKD patients and non-ADPKD patients throughout the examination day (*p* = 0.882 in Period 1, *p* = 0.333 in Period 2, *p* = 0.622 in Period 3 and *p* = 0.450 in Period 4).

### Vasoactive hormones

Table 4 shows vasoactive hormones in plasma.

**Table 4:** Plasma concentrations of vasopressin (p-AVP), renin (PRC), aldosterone (p-Aldo), angiotensin II (p-AngII), and plasma osmolality (p-Osm) during a water deprivation test in patients with adult dominant polycystic kidney disease (ADPKD), patients with non-ADPKD kidney disease (Non-ADPKD), and healthy control subjects (Controls). Urine was collected in four periods. Period 1 (twelve hours): from 0730 PM the day before arrival to the laboratory to 0730 AM on the examination day. Period 2 (one hour): 0730 to 0830 AM. Period 3 (two hours): 0830–1030 AM. Period 4 (two hours): 1030–1230 AM. Blood samples were taken at the end of periods 2, 3 and 4

	End of Period 2 08.30 AM	End of Period 3 10.30 AM	End of Period 4 12.30 AM
P-AVP (pg/ml)			
ADPKD	0.5 ± 0.4	0.6 ± 0.4	0.6 ± 0.4
Non-ADPKD	0.6 ± 0.4	0.6 ± 0.3	0.6 ± 0.3
Controls	0.4 ± 0.3	0.4 ± 0.3	0.5 ± 0.3
Comparison within groups	0.753		
Comparison between groups	0.317		
PRC (pg/ml)			
ADPKD	7.4 [4.3;8.8]#	5.4 [4.1;8.6]#	6.0 [3.6;8.7]#
Non-ADPKD	12.9 [5.8;26.4]	11.4 [4.9;26.4]	10.6 [4.8;24.8]
Controls	7.1 [5.9;10.4]	7.4 [4.7;9.7]	6.4 [4.8;8.7]
Kruskal-Wallis test	0.050	0.054	0.079
P-AngII (pg/ml)			
ADPKD	5.5 ± 4.0	5.0 ± 3.1	5.7 ± 3.5
Non-ADPKD	6.6 ± 3.6	6.1 ± 2.7	6.2 ± 3.2
Controls	5.7 ± 2.4	6.0 ± 3.3	5.4 ± 3.0
Comparison within groups	0.348		
Comparison between groups	0.715		
P-Aldo (pmol/l)			
ADPKD	324 [203;478]*	305 [218;484]*	273 [222;385]*
Non-ADPKD	257 [201;314]*	230 [179;380]*	213 [172;334]*
Controls	145 [126;223]	120 [99;157]	101 [95;154]
Kruskal-Wallis test	< 0.001	< 0.001	< 0.001
P-Osm (mosmol/kg)			
ADPKD	294 ± 4	293 ± 4	293 ± 4
Non-ADPKD	296 ± 8	296 ± 8	295 ± 7
Controls	293 ± 4	293 ± 4	292 ± 4
Comparison within groups	0.651		
Comparison between groups	0.236		

A General Linear Model with Greenhouse-Geisser correction was used for comparison within and between the three groups. Values represent mean ± SD or median with 25 and 75% percentiles in brackets * = p < 0.05 between ADPKD or non-ADPKD- patients and healthy controls. # = *p* < 0.05 between ADPKD and non-ADPKD- patients

PRC was lower in ADPKD patients compared to non-ADPKD patients throughout the examination day (*p* = 0.021 in Period 2, *p* = 0.027 in Period 3 and *p* = 0.031 in Period 4). There was no difference between healthy controls and ADPKD or non-ADPKD patients (*p* > 0.05). P-Aldo was higher in ADPKD and non-ADPKD patients compared to healthy controls throughout the examination day (*p* < 0.05). There was no difference between ADPKD and non-ADPKD patients (*p* = 0.221 in Period 2, *p* = 0.171 in Period 3 and *p* = 0.121 in Period 4). P-AngII did not change during the examination day (*p* = 0.348), and there were similar p-AngII-levels in the three groups (*p* = 0.715).

### Systemic haemodynamics

Table 5 shows systolic bBP, diastolic bBP, mean blood pressure, heart rate, systolic cBP, diastolic cBP, AIx and PWV.

**Table 5:** Hemodynamics during a water deprivation test in patients with adult dominant polycystic kidney disease (ADPKD), patients with non-ADPKD kidney disease (Non-ADPKD), and healthy control subjects (Controls)

	08.30 AM-09-30 AM	09.30 AM–10.30 AM	10.30 AM–11.30 AM	11.30 AM-12.30 PM
Systolic bBP (mm Hg)				
ADPKD	129 ± 9*#	131 ± 9*#	133 ± 11*#	131 ± 12*
Non-ADPKD	122 ± 14*	124 ± 16*	125 ± 16*	126 ± 18*
Controls	113 ± 12	117 ± 12	118 ± 14	120 ± 16
Comparison within groups	0.282			
Comparison between groups	0.004			
Diastolic bBP (mm Hg)				
ADPKD	83 ± 9*#	84 ± 9*#	84 ± 9*#	84 ± 10*#
Non-ADPKD	77 ± 9	76 ± 10	77 ± 11	77 ± 11
Controls	74 ± 9	74 ± 10	76 ± 11	76 ± 11
Comparison within groups	0.394			
Comparison between groups	< 0.001			
MAP (mm Hg)				
ADPKD	104 ± 8*#	106 ± 9*#	107 ± 9*#	106 ± 10*#
Non-ADPKD	98 ± 11*	98 ± 12	99 ± 13	100 ± 14
Controls	92 ± 10	94 ± 11	95 ± 12	96 ± 13
Comparison within groups	0.268			
Comparison between groups	< 0.001			
HR (beats/ min)				
ADPKD	60 ± 10*	62 ± 11*	62 ± 11*	64 ± 11*
Non-ADPKD	59 ± 8	60 ± 7*	61 ± 8*	62 ± 8*
Controls	57 ± 6	57 ± 6	58 ± 7	59 ± 7
Comparison within groups	0.250			
Comparison between groups	0.029			
Systolic cBP (mmHg)				
ADPKD	119 ± 11*#	122 ± 11*#	123 ± 13*#	122 ± 14*
Non-ADPKD	112 ± 14*	114 ± 15	115 ± 15	117 ± 17
Controls	106 ± 12	110 ± 13	111 ± 13	113 ± 15
Comparison within groups	0.195			
Comparison between groups	< 0.001			
Diastolic cBP (mm Hg)				
ADPKD	84 ± 9*#	85 ± 9*#	86 ± 9*#	85 ± 10*#
Non-ADPKD	78 ± 10	76 ± 10	78 ± 11	78 ± 12
Controls	74 ± 10	75 ± 11	76 ± 10	76 ± 11
Comparison within groups	0.710			
Comparison between groups	< 0.001			
AIx (%)				
ADPKD	31 ± 13	28 ± 13	31 ± 13	28 ± 13
Non-ADPKD	31 ± 16	31 ± 16	29 ± 17	29 ± 15
Controls	31 ± 12	32 ± 14	29 ± 13	28 ± 14
Comparison within groups	0.645			
Comparison between groups	0.903			
PWV (m/s)				
ADPKD	8 ± 2	8 ± 2	8 ± 2	8 ± 2
Non-ADPKD	8 ± 2	8 ± 2	8 ± 2	8 ± 2
Controls	8 ± 2	8 ± 2	8 ± 2	8 ± 2
Comparison within groups	0.124			
Comparison between groups	0.819			

A General Linear Model with Greenhouse-Geisser correction was used for comparison within and between the three groups. Values represent mean ± SD or median with 25 and 75% percentiles in brackets * = p < 0.05 between ADPKD or non-ADPKD patients and healthy controls. # = p < 0.05 between ADPKD and non-ADPKD patients

Systolic bBP were higher in ADPKD and non-ADPKD patients compared to healthy controls (p < 0.05). Systolic bBP was also increased in ADPKD patients compare to non-ADPKD patients, except during Period 4 (*p* = 0.009 in Period 1, *p* = 0.015 in Period 2, *p* = 0.005 in Period 3 and *p* = 0.057 in Period 4). Systolic cBP showed the same pattern regarding to ADPKD patients in comparison to non-ADPKD patients and healthy controls. However, non-ADPKD patients only had increased systolic cBP compared to healthy controls during Period 1 (*p* = 0.038). Mean BP, diastolic bBP and diastolic cBP were increased in ADPKD patients compare to non-ADPKD patients and healthy controls throughout the examination day (*p* < 0.05).

Heart rate was slightly increased in both group of patients compared with the controls (p < 0.05).

AIx and PWV did not change during the examination day (*p* = 0.645 and *p* = 0.124,respectively) and there were similar AIx and PWV in the three groups (*p* = 0.903 and *p* = 0.819,respectively).

## Discussion

The present paper reports urine concentration ability, tubular function, vasoactive hormones and systemic hemodynamics in ADPKD patients compared to patients with similar degree of impairment in renal function (non-ADPKD patients), and healthy controls during seventeen hours of water deprivation.

The main findings were that urine concentration ability was reduced to the same extent in patients with ADPKD and non-ADPKD chronic kidney diseases with the same level of renal function compared to healthy controls. In addition, the lower urine excretion rates of AQP2 and ENaC suggest that the underlying mechanism may be a reduced tubular response to vasopressin and aldosterone.

### Renal water excretion

During the first 12 h of the concentration test (Period 1), we did not measure any significant difference in u-Osm, UO, C_H2O_ and AQP2 excretion rate between the 3 groups. During the following 5 h (Periods 2–4), u-Osm, UO and AQP excretion rate were the same in ADPKD and non-ADPKD, but significantly lower than in controls, except for UO, which was increased compared to healthy controls. Apparently, a plateau in u-Osm was reached in Period 2 without further significant changes during the following periods. The maximum u-Osm in the healthy controls was around 760 mosmol/kg. This is lower than expected in younger healthy subjects, but urine concentrating ability decreases with age, and our control group of healthy subjects had an average age of 57 years, which explains the u-Osm level in the control group. Vasopressin is responsible for the final regulation of renal water excretion, which occurs in the distal part of the nephron. Vasopressin is released by increased osmotic activity in plasma, and the hormone stimulates opening of the aquaporin2 water channels in the principal cells in the distal part of the nephron via a cascade of intracellular reactions. This facilitates water absorption. Aquaporin 2 is excreted in the urine as extracellular vesicles, and AQP2 is localized predominantly to urinary exosomes with preserved water channel activity [25]. The amount of AQP2 in exosomes released from the collecting duct cells is physiologically regulated, and exosomal AQP2 closely reflects cellular expression [26]. When urine is stored in a deep freezer, the membranes of the extracellular vesicles are disrupted, which allow AQP2 antibodies to bind to their epitopes located inside the extracellular vesicles [27]. Our measurement of u-AQP2 reflects the activity of the transport via the aquaporin water channels. The results showed that urine concentration ability was impaired in both groups of patients with kidney diseases. In addition, the lower level of AQP2 excretion rate demonstrated a tubular defect or a reduced tubular responsiveness in patients, which is in accordance with an earlier study [9]. We could not falsify the 0-hypothesis that ADPKD patients have same urine concentrating capacity as patients with other types of chronic kidney diseases. – at least at a level of GFR around 60–70 ml/min. In good agreement with the present study, Meijer et al. measured a decreased maximal u-Osm in ADPKD compared to healthy controls after intramuscular injection of desmopressin [15]. Contrary to our study, Zittema et al. measured decreased maximal u-Osm in patients with ADPKD compared with IgA-nephropathy after desmopressin injection. This suggested a different concentration ability depending on primary kidney disease [17]. In a previous study, we measured similar u-Osm in chronic kidney disease, when ADPKD was excluded. Most likely, the discrepancies in results between the studies can be attributed to differences in eGFR, in test conditions such as duration of the test and degree of water deprivation, methods used, i.e. thirst or desmopressin injection, and primary kidney disease. Overall, patients with kidney disease and moderate to severe reduction in renal function have a decrease in urine concentration ability. This abnormality can be attributed to reduced tubular capacity to decrease water excretion, which in the present study is documented by the lower AQP2 excretion rate than in healthy controls.

During water deprivation, body weight decreased to the same extent in all three groups around 2.2 to 2.4 kg on average, which documented that the patients and controls had thirsted. However, p-AVP, p-Osm and C_H2O_ did not change significantly during water deprivation and did not differ between the groups. Increased or unchanged levels of p-AVP, p-Osm and C_H2O_ have previously been reported during urine concentration tests depending on the degree of renal impairment in patients studied [8, 9, 16, 17]. In our study, patients had not severely impaired renal function, which can explain the lack of deviation from the control group regarding these variables. In addition, a prolonged water deprivation test might have demasked differences between the two groups of patients.

The activity in the osmoregulatory system can be measured by p-AVP, but plasma levels of copeptin have also been used in recent years. Pre-pro-AVP is proteolytically cleaved into vasopressin, neurophysin II and copeptin, and the three substances are stored in secretory granules in the posterior pituitary. They are released on osmotic and non-osmotic stimuli and copeptin and vasopressin are released in equimolar amounts into the circulation. Thus, copeptin can be used as a surrogate vasopressin marker. Since copeptin is more stable in plasma than vasopressin, some researchers have used copeptin to evaluate the activity in the osmoregulatory system. However, when blood samples for vasopressin measurement are drawn, prepared and plasma stored under well-defined conditions, p-AVP correctly reflects the activity in the osmoregulatory system both during normal physiological conditions and during disturbances in fluid balance [28–30] .

### Renal sodium excretion

Zittema et al. measured an increase in u-Na in ADPKD patients during a water deprivation test, but there was no change in IgA patients [17]. In the present study, FE_Na_ was the same throughout the examination day in ADPKD and non-ADPKD patients, but FE_Na_ was increased compared to healthy controls as expected. We measured a lower ENaC excretion rate in both ADPKD, and non-ADPKD patients compare to healthy controls during urine concentration test. This suggests a defect in tubular function with a reduced response to aldosterone, which was increased in both groups of patients compared to the control group. It is in good agreement with previous experimental studies of collecting duct principal cells in mice [31].

### Renal function

Clearance of creatinine was the same during the consecutive periods of the urine concentration test in the two groups of patients and the healthy controls. However, albumin excretion rate was higher in the non-ADPKD patients during the test than in ADPKD and controls. This could indicate a lower tubular albumin absorption in non-ADPKD, which presumably reflects different types of tubular lesion among the two groups of patients. Another explanation could be a more severe glomeruli injury in the non-ADPKD patients compare to ADPKD patients. These results are in agreement with the findings of Zittema et al., who measured an increase in u-Alb in IgA patients but no change in ADPKD patients [17].

### Vasoactive hormones

In the present paper, we measured a higher level of p-Aldo in both ADPKD and non-ADPKD compared to healthy controls, which is in agreement with other studies [32, 33]. The renin-angiotensin-aldosterone system regulates blood pressure and renal sodium excretion via angiotensin’s vasoconstrictor effect on the vascular bed, and via aldosterone stimulation of sodium absorption in the distal part of the nephron. The effect of aldosterone is mediated by an effect on the epithelial sodium channels. Our measurement of u-ENaC reflects the activity of the transport via these channels. As aldosterone regulates ENaC using the mineralocorticoid receptor in the principal cells, it could be expected that u-ENaC also was higher in the two groups of patients. However, u-ENaC was lower in patients than controls. The most likely explanation is a lack of tubular responsiveness to aldosterone caused by the primary kidney disease. The enhanced PRC in non-ADPKD patients compared to ADPKD patients and healthy controls is in accordance with a previous study [34].

### Systemic hemodynamics

During the water deprivation test, ADPKD patients had a slightly higher systolic and diastolic bBP and cBP compare to non-ADPKD patients and healthy controls. This was in spite of the fact that more patients in the ADPKD group received more antihypertensive therapy. However, we do not think that these small difference in blood pressure level could explain any differences in effects variables in the present study.

## Strengths and limitations

The match regarding age, gender and renal function is a strength in our study as well as the uniform procedure of the concentration test, and its’ length. Patients received antihypertensive treatment during the study because we did not find it ethically justified to discontinue antihypertensive medication. It was standardized to only metoprolol and/or amlodipine, and a possible influence on the results is estimated to be absent or minimal during the concentration test. We did not measure blood pressure at the start of the water deprivation test, but it was controlled regularly during the weeks before the patients were studied, and many times during the last four periods of water deprivation. It is a weakness of the study that the first part of the water deprivation test took place at home and not in hospital. Thus, a fluid intake could not completely be excluded. However, the weight loss documented the patients had thirsted. The wide range of impaired kidney functions, from CKD I-IV may also be a limitation to the interpretation of the results. It can be speculated, whether a significant difference in other effect variables might be detected between the two groups of patients, if the groups have been larger, but we calculated the size of the groups based on the best available information.

## Conclusion

Urine concentration ability was reduced to the same extent in patients with ADPKD and other chronic kidney diseases with the same level of renal impairment compared to healthy controls. In addition, the lower urine excretion of AQP2 and ENaC suggest that the underlying mechanism may be reduced tubular response to vasopressin and aldosterone. We could not falsify the 0-hypothesis that ADPKD patients have same urine concentrating capacity as patients with other types of chronic kidney diseases.

## Data Availability

The protocol and datasets used and/or analysed during the current study are available from the corresponding author on reasonable request.

## Abbreviations

ALAT: alanine-amino-transaminase
AIx: augmentation index
ADPKD: Autosomal dominant polycystic kidney disease
AQP2: aquaporin-2
bBP: brachial blood pressure
cBP: central blood pressure
C_Cr_: creatinine clearance
C_H2O_: free water clearance
CKD: chronic kidney disease
eGFR: estimated glomerular filtration rate
ENaC: epithelial sodium channel
FE_Na_: fractional excretion of sodium
HbA1c: glycosylated hemoglobin A1c
Non-ADPKD: patients with chronic kidney disease except ADPKD
MAP: mean arterial pressure
P: *p*-value
p-Aldo: plasma aldosterone
p-AngII: plasma angiotensin
p-AVP: plasma vasopressin
P-Osm: plasma osmolality
PRC: plasma renin concentration
PTH: parathyroid hormone
PWV: pulse wave velocity
TSH: thyroid-stimulating hormone
UO: urine output
U-Osm: urinary osmolality
